# Nightmare and Abnormal Dreams: Rare Side Effects of Metformin?

**DOI:** 10.1155/2018/7809305

**Published:** 2018-01-17

**Authors:** Theo Audi Yanto, Ian Huang, Felicia Nathania Kosasih, Nata Pratama Hardjo Lugito

**Affiliations:** ^1^Internal Medicine Department, Faculty of Medicine, Universitas Pelita Harapan, Jalan Jendral Sudirman Boulevard No. 20, Lippo Karawaci, Tangerang, Banten 15811, Indonesia; ^2^Faculty of Medicine, Universitas Pelita Harapan, Jalan Jendral Sudirman Boulevard No. 20, Lippo Karawaci, Tangerang, Banten 15811, Indonesia

## Abstract

**Background:**

Metformin is widely known as an antidiabetic agent which has significant gastrointestinal side effects, but nightmares and abnormal dreams as its adverse reactions are not well reported.

**Case Presentation:**

Herein we present a case of 56-year-old male patient with no known history of recurrent nightmares and sleep disorder, experiencing nightmare and abnormal dreams directly after consumption of 750 mg extended release metformin. He reported his dream as an unpleasant experience which awakened him at night with negative feelings. The nightmare only lasted for a night, but his dreams every night thereafter seemed abnormal. The dreams were vivid and indescribable. The disappearance and occurrence of abnormal dreams ensued soon after the drug was discontinued and rechallenged. The case was assessed using Naranjo Adverse Drug Reaction (ADR) probability scale and resulted as probable causality.

**Conclusion:**

Metformin might be the underlying cause of nightmare and abnormal dreams in this patient. More studies are needed to confirm the association and causality of this findings.

## 1. Introduction

Metformin is widely known as oral antidiabetics with long history of effectiveness and safety [1]. Despite the fact that the efficacy of metformin offers wide range of benefits beyond its glycemic control, physicians have always been well-aware of its adverse effects [1, 2]. Metformin-induced gastrointestinal symptoms and the debatable lactic acidosis are universally recognized by the clinicians [2, 3]; however its side effects in inducing nightmare and abnormal dreams are not well reported. Herein, we report a probable case of metformin-induced nightmare and abnormal dreams.

## 2. Case Report

A 56-year-old male with no previous history of depression and sleep disorder experienced nightmare and abnormal dreams after consumption of 750 mg extended release metformin. He described his dream as bizarre which awakened him at night with negative feelings. The nightmare only lasted for a night, but the dreams every night thereafter seemed abnormal. He reported that the dreams did not affect his quality of sleep except for the first night. He had been diagnosed with type 2 diabetes mellitus (T2DM) 5 years ago and managed to control his blood glucose level by dietary modification. He concurrently took atorvastatin and candesartan for his hypertension and dyslipidemia. The Pittsburgh Sleep Quality Index (PSQI) and Hamilton Psychiatric Rating Scale for Depression (HAM-D17) showed very good sleep quality and negative results for depressive symptoms. He stated that he was happy with his life and working environment. Physical examination showed that the patient was overweight but other examinations were otherwise unremarkable. Liver enzymes, blood creatinine and urea were within normal limits. His A1C hemoglobin level was 7.6%.

In attempt to evaluate the causality of the nightmare and abnormal dreams, the patient agreed to temporarily discontinue and rechallenge atorvastatin, candesartan, and metformin, respectively. Every single drug was consumed for four consecutive days and then was changed to another drug. On the night when metformin was reintroduced, the abnormal dreams ensued. Although it did not affect his overall sleep quality and daily life, his abnormally vivid dreams continued for 4 following nights as the metformin was rechallenged.

After the extensive potential benefits of metformin were explained, the patient agreed to continue taking metformin 750 mg per day for the first week and metformin was uptitrated to 1500 mg per day in a 3-month follow-up plan. Three months after the initial visit, he reported that he still experienced abnormal dreams every night with no interference of daily activities. He noticed that the symptoms temporarily worsen when the dose was increased and felt better if he consumed metformin in the morning. He also occasionally experienced a mild metallic taste and bloating. His A1C hemoglobin level on the follow-up visit was 6.5%. The patient still reported the same abnormal dreams every night even after 6 months of using metformin. Both his global PSQI score and HAM-D17 were 0, and A1C hemoglobin level was 6.7% on the last visit.

## 3. Discussion

Dreaming is a complex cognitive process in human central nervous system during sleep that can be affected by wide variety of variables, including medical, psychological, sleep, and social factors [4]. Despite the fact that the precise pathophysiology of disordered dreaming is largely unknown and possibly involves complex neurochemical process, drugs that affected neurotransmitters and rapid eye movement (REM) sleep have been shown to cause nightmares [5]. Moreover, sleep disorder and disordered dreaming were commonly found in patients with T2DM with overall prevalence as high as 50% [6]. The occurrence of bad dreams and poor sleep quality among patients with T2DM were associated with poor glycemic control and duration of disease [7, 8].

Metformin is one of the oldest oral antidiabetic drugs that has excellent profile of its efficacy and safety since its widespread use in 1978 [1, 2, 9–12]. While there were many numerous reviews and meta-analyses of metformin adverse effects, only a brief review and a case report mentioned the possible causality of metformin-induced nightmare and abnormal dreams [13, 14]. Nonformal accounts of these occurrence were reported from* eHealthMe.com, *a website which is used as patient self-reporting adverse drug effects and primarily connects with the data from US Food and Drug Administration (FDA), with possible overall prevalence of 0.17% and 0.23% over 200,000 people who routinely took metformin [15, 16].

This present case was carefully managed in a fashion to prove the causality. After the drugs rechallenge test, the case was then assessed with Naranjo ADR scale which resulted as probable causality for metformin-induced nightmare and abnormal dreams (total score of 6). The possibility of poor sleep quality and depressive disorders as confounding factors of nightmare and abnormal dreams were excluded by normal results of the PSQI and HAM-D17. While the T2DM itself might be the underlying cause of the nightmare and abnormal dreams in this patient, it could not entirely explain why the specific symptoms straightly and consecutively ensued directly after the metformin was consumed. The aggravation and amelioration of the symptoms were dose-dependent and the changed time of administration might have interfered with the dose-response relationship and drug concentration level in inducing the side effects.

Cerebral blood glucose levels during the night seems to play a key role in explaining the occurrence of nightmare and abnormal dreams in this case [13]. Nocturnal hypoglycemia is often clinically asymptomatic, but it could cause problems related to abnormal dreams and poor sleep quality in some individuals [17]. Even though it is the most plausible cause in this case, there are some theoretical issues worthy of mentioning. Metformin monotherapy in its therapeutic dose along with the absence of strenuous exercise, fasting, alcoholism, old age, malnutrition, and other medical comorbidities is very unlikely to cause hypoglycemia [1, 11, 18]. Furthermore, the absence of fatigue, negative mood changes, and well-being associated with nocturnal hypoglycemia on the following day [17, 19] may provide us with a clue of another possible mechanism of abnormal dreams associated with metformin.

## 4. Conclusion

Nightmares and abnormal dreams may be the rare side effects of metformin. Studies are needed to confirm the association and causality of these findings.
